# Safety, efficacy and pharmacokinetic evaluations of a new coated chloroquine tablet in a single-arm open-label non-comparative trial in Brazil: a step towards a user-friendly malaria vivax treatment

**DOI:** 10.1186/s12936-016-1530-0

**Published:** 2016-09-17

**Authors:** Dhelio Pereira, André Daher, Graziela Zanini, Ivan Maia, Lais Fonseca, Luciana Pitta, Rosilene Ruffato, Paola Marchesini, Cor Jesus Fontes

**Affiliations:** 1 Tropical Medicine Research Center of Rondônia (CEPEM), Porto Velho, Rondônia Brazil; 2 Laboratory of Parasitology, National Institute of Infectious Disease, Oswaldo Cruz Foundation (FIOCRUZ), Rio de Janeiro, Brazil; 3 Federal University of Rondônia (UNIR), Porto Velho, Rondônia Brazil; 4 Institute of Drug Technology (Farmanguinhos), Oswaldo Cruz Foundation (FIOCRUZ), Rio de Janeiro, Brazil; 5 Vice-presidency of Research and Reference Laboratories, Oswaldo Cruz Foundation (FIOCRUZ), Rio de Janeiro, Brazil; 6 Liverpool School of Tropical Medicine, Liverpool, UK; 7 Laboratory of Pharmacokinetics (Sefar), Oswaldo Cruz Foundation (FIOCRUZ), Rio de Janeiro, Brazil; 8 National Programme of Malaria Control, Ministry of Health, Brasília, Brazil; 9 Julio Müller Hospital, Federal University of Mato Grosso, Cuiabá, Brazil

**Keywords:** Malaria, *Plasmodium vivax*, Antimalarial treatment, Chloroquine, Pharmacokinetics, Adherence, Clinical trial

## Abstract

**Background:**

Malaria remains a major public health problem, with half the world population at risk of contracting malaria. The effects of *Plasmodium vivax* on prosperity and longevity have been highlighted in several recent clinical case reports. The first line of vivax treatment drugs has seen no radical innovation for more than 60 years. This study introduces a subtle incremental innovation to vivax treatment: a chloroquine and primaquine co-blister. The co-blister includes a new chloroquine formulation incorporating coated tablets to mask the drug’s bitter taste and user-friendly packaging containing tablets of each drug, which may improve patient adherence and facilitate the appropriate use of the drugs. This new formulation will replace the non-coated chloroquine distributed in Brazil.

**Methods:**

Patients were orally treated with 150 mg coated chloroquine tablets for 3 days: an initial 450 mg dose, followed by two 300 mg doses. The patients were treated concomitantly with two 15 mg primaquine tablets for 7–9 days, according to their weight. The primary objective of this study was to prove parasitological and clinical cure rates above 90 % by day 28.

**Results:**

This single-arm open-label non-comparative trial was conducted according to the WHO recommended methodology for the surveillance of anti-malarial drug efficacy in the Brazilian Amazon. On day 28, the parasitological and clinical response was adequate in 98.8 % of patients (CI 95 % 93.4–100 %). The success rate on day 3 was 100 %, and the cumulative success rate by day 28 was 98.8 % (CI 95 % 91.7–99.8 %). There were no serious adverse events, with most adverse events classified as mild. The pharmacokinetic parameters of chloroquine analysed in whole blood dry spot samples showed mean (coefficient of variation) Cmax and AUC_0–t_ values of 374.44 (0.35) and 3700.43 (0.36) ng/mL, respectively.

**Discussion:**

This study reports an appropriate safety and efficacy profile of a new formulation of coated chloroquine tablets for vivax malaria treatment in the Brazilian Amazon. The cure rates meet the WHO efficacy criteria, supporting current Brazilian guidelines and the use of the formulation for vivax malaria treatment. Nevertheless, further studies should be conducted to address adherence and the effectiveness of the formulation.

*Trial registration* RBR-77q7t3-UTN: U1111-1121-2982. Registered 10th May 2011

**Electronic supplementary material:**

The online version of this article (doi:10.1186/s12936-016-1530-0) contains supplementary material, which is available to authorized users.

## Background

Malaria remains a major public health problem. Despite many advances in the past 15 years, 3.2 billion people are at risk of contracting malaria, which represents half of the population of the world. According the World Health Organization (WHO), in its 2015 World Malaria Report, the number of malaria cases decreased from an estimated 262 million in 2000 (range 205–316 million) to 214 million in 2015 (range 149–303 million), a decline of 18 % [1, 2].

*Plasmodium falciparum* has been the primary target of malaria drug development for the past 15 years. The high mortality, the emergence of drug resistance and the record high number of cases in Africa justify this approach. Although falciparum malaria is still a major public health problem, during the past decade, key achievements have dramatically changed its epidemiological profile, such as new rapid diagnosis tests (RDT) and new treatments comprising fixed dose combinations of artemisinin derivatives and other anti-malarial drugs. In Brazil, the incidence ratio of falciparum/vivax has considerably changed since artemisinin-based combination therapy (ACT) has been recommended as the first-line treatment. In 1988, *Plasmodium vivax* and *P. falciparum* had incidence rates of 50 % each. In 1990, *P. falciparum* corresponded to 44.3 % of cases; in 2009, 3 years after ACT implementation, *P. vivax* represented 84 % of the recorded cases, whereas *P. falciparum* represented only 16 % [3]. In 2014, 143,552 cases of malaria were reported. Although the falciparum/vivax ratio was not constant between the years of 2012 and 2013 [4], *P. vivax* infections accounted for 84 % of cases in 2014. The total number of cases declined by 19 % compared to 2013. The overall number of cases is currently the lowest it has been for the past 35 years in Brazil [5].

The importance of *P. vivax* has been highlighted based on its effects on prosperity and longevity [6, 7], and many severe and fatal clinical cases reports have been published [8–13]. In 2014, *P. vivax* was the most frequent cause of hospitalization (63 %) and death (11/23) due to malaria in the Brazilian Amazon region [5]. In addition to new cases, 39,279 possible recurrence cases were reported in 2012 [4], among which the recurrences were due to re-infection, resistance or non-compliance to the treatment [14].

In addition, various barriers are known to impair transmission control as the early appearance of gametocytes and relapses from hypnozoites. In 2009, Guerra et al. [15] estimated that 2.85 billion people were exposed a risk of vivax infection.

The first line drug treatment for vivax malaria has been used for more than 60 years with no radical innovation. To address the lack of research progress on this most neglected disease [16], an incremental innovation to *P. vivax* treatment was developed in this study: a chloroquine and primaquine co-blister. This approach is a formulation and packaging improvement of the current vivax treatment recommended by the Brazilian Ministry of Health. The co-blister of chloroquine and primaquine was developed assuming that a user-friendly primary package containing both tablets of concomitant use may improve adherence [17]. The co-blister preliminary design is provided in Additional file 1. Stratifying the primary package according patient age ranges may facilitate the appropriate use of the drugs [18] especially in the majority of drug distribution settings in Brazil where the drugs dispensers are communitarian health agents [19]. In Brazil, vivax treatment has five weight ranges, varying from 17 to 26 tablets in three different tablet concentrations. Although health agents may have their own prescription strategies, difficulties remain regarding ensuring the correct posology when ten loose tablets are prescribed, especially in households in which illiterate people reside or with more than one patient.

The new user-friendly primary polyvinylidene chloride (PVDC) package of coated chloroquine and primaquine may replace the non-coated chloroquine packed in paper strips that have been distributed in Brazil. The co-blister includes a new chloroquine formulation of coated tablets. Coating the chloroquine masks the bitter taste of the drug and may improve treatment adherence. Although it was not studied in this population, bitter taste is known as a factor that may impair adherence [20]. In this study, the evaluation of improvements upon treatment was focused on the formulation rather than the dosing. The primary objective of this study was to prove parasitological and clinical cure rates of the new formulation above 90 % by day 28. This paper presents the safety and efficacy results of this new formulation. These results represent the first step towards the introduction of a user-friendly co-blister of chloroquine and primaquine to treat malaria vivax in Brazil. Nevertheless, further studies should be conducted to address adherence and effectiveness of the new chloroquine formulation and the co-blister.

## Methods

### Ethics and regulatory statement

The clinical study protocol and informed consent were reviewed and approved by the Ethics Committee at the Tropical Medicine Research Center of Rondônia (Nº 01/11 CEP/CEPEM e Nº 0001.0.046.000-11 CAAE-SISNEP). The Brazilian National Council on Ethics in Research (CONEP), Ministry of Health, accredits this committee. The clinical study was conducted in accordance with the Helsinki Declaration, Good Clinical Practice [21] and the Brazilian National Health Council (CNS) resolutions 196/1996 and 466/2011. The study is registered with the Brazilian Register of Clinical Trials (RBR-77q7t3-UTN: U1111-1121-2982). The protocol was also approved by the National Regulatory Agency, ANVISA (CE No. 188/2012-0896111125), and this study was conducted according the Good Clinical Practice (GCP) [22]. During the study, visits were conducted to ensure GCP adherence. Written informed consent was obtained for every subject prior to enrolment. If the study subject was illiterate, an impartial third party witnessed the informed consent process. All subjects were informed of the nature of the trial and the possible risks bound to it and that they were free to withdraw their consent to participate at any time. The investigators and study staff ensured confidentiality of all records.

### Study population

Patients with vivax malaria mono-infection willing to participate were included in the study after signing the informed consent form if they met the following inclusion criteria: aged between 18 and 70 years; weight between 50 and 90 kg, *P. vivax* infection confirmed by microscopy, asexual parasite count >250/μL; either axillar temperature ≥37.5 °C or a history of fever during the past 48 h; the ability to swallow tablets; and the availability to attend the study follow-up visits. Patients who met one of the following criteria were not eligible to participate: prior malaria treatment in the past 63 days; signs of severe malaria; either other febrile conditions or chronic disease (such as severe cardiac, hepatic or renal disorders and AIDS); the use of any medication that interferes with anti-malarial pharmacokinetics; an intolerance to chloroquine or primaquine; a known glucose-6-phosphate deficiency; pregnancy confirmed by human chorionic gonadotropin (hCG) in their urine or breastfeeding; or among the indigenous population.

### Study design and drug administration procedures

This single arm open-label non-comparative trial was conducted according the WHO recommendations [23] at the Tropical Medicine Research Centre of Rondônia (CEPEM). This centre is located in the Amazon Region of Brazil. The demonstration of at least a 90 % cure rate is considered by the WHO to be satisfactory evidence of efficacy to support the National Malaria Control Programme treatment guidelines [23]. The sample size was calculated with an expected failure rate of 5 %. To achieve a precision level of 5 % and a possible loss of 20 % of participants by the follow-up, 88 patients were included.

Eligible patients were treated in accordance with the Brazilian Ministry of Health recommendation [24]. All patients who were included were orally treated with 150 mg coated chloroquine tablets (Farmanguinhos—Fiocruz, batch number 11090655) for 3 days. On the first day, a higher dose of chloroquine was administered (450 mg) followed by 2 days of 300 mg doses per day. Patients were also concomitantly treated with two tablets of primaquine 15 mg (Farmanguinhos—Fiocruz, batch numbers 12010038; 13030282) for 7, 8 or 9 days according three weight ranges (≥50–69; 70–79; 80–90 kg), respectively. The total primaquine dose was never lower than 3 mg/kg or higher than 4.2 mg/kg. An illustrative table is provided as an additional file (Additional file 2).

Chloroquine and primaquine administrations were supervised during the first 3 days. The first dose was taken after diagnosis, and the following doses were administered between 8 and 10 a.m. If the patient vomited within 30 min after a dose, the same dose was administered again. Treatment adherence to primaquine between days 4 and 7 was assessed by inquiring with the patients at the day 7 follow-up visit. The patients were also asked about the use of concomitant therapies at every visit and recorded.

### Safety and efficacy evaluations

Patients were evaluated on the day of enrolment and on days 1, 2, 3, 7, 14, 21 and 28 after the study period. The scheduled study procedures included a complete clinical anamnesis, a physical exam and a urinalysis at enrolment and an assessment of the most frequent clinical signs of malaria and adverse events (AEs) at all the follow-up visits. Additionally, blood samples were collected for parasite counts (0, 2, 3, 7, 14, 21 and 28 days), haemoglobin and haematocrit evaluations (0, 14 and 28 days), genotyping (0, 7, 14, 21 and 28 days) and chloroquine pharmacokinetic analyses (0, 3, 7, 14, 21 and 28 days).

The primary efficacy endpoint was the proportion of the population with an adequate clinical and parasitological response; otherwise, it was classified either as early treatment failure or late treatment failure.

Both per protocol (PP) and intention-to-treat (ITT) analyses were conducted, for which the PP was the primary analysis, and the ITT analysis was performed to confirm the results. The PP population excluded any participant associated with a protocol violation or who was lost by the follow-up visit at the primary endpoint on day 28, whereas the ITT population included any violation or loss by the follow-up as parasitological and clinical failure. The cumulative success rate by day 28, corresponding to the probability of remaining parasite-free at day 28, was calculated using a Kaplan Meyer survival curve. The absolute number of patients who presented symptoms and parasitaemia 72 h after treatment were defined as early treatment failures. Demographic data were presented for both populations. All the data were summarized as frequencies and percentages for categorical variables and as the means ± standard deviations (SD) for quantitative variables.

The safety analysis was conducted in the ITT population describing the frequency of AEs and stratifying the AEs among the follow categories: serious AEs, AEs leading to treatment suspension, and AEs corresponding to causalities described as doubtful, unlikely, possible or probable. The mean haemoglobin and haematocrit results at baseline and at the follow-up visits are also presented.

The patients were encouraged to seek unscheduled assessments if any AE was suspected. All clinical or laboratory abnormalities were categorized as grade I to IV according to the common terminology criteria for adverse events (CTCAE) of the National Cancer Institute [25]. Any suspected serious AE as defined by good clinical practice was reported to the sponsor and the Ethical Review Committee. Any disorder recorded at the inclusion anamnesis or the recurrence of the disorder were not recorded as an AE. However, any exacerbation of a previous disorder, either in frequency or intensity, was described as an AE. Known drug-related events were recorded as an AE, even if it could be classified as a malaria symptom.

The parasitological densities were estimated using Giemsa-stained blood slides at a magnification of 1000× as the WHO recommended methodology for the surveillance of anti-malarial drug efficacy [23]. Two trained microscopists provided independent results, and the final densities corresponded to the average of the two independent assessments. A third microscopist evaluated discordant results pertaining to the presence or absence of parasites, the diagnosis of different species, or the report of a parasite density greater than 50 % in the same slide. In this case, the final densities corresponded to the average of the two closest counts. Negative results were issued only after the evaluation of 1000 leucocytes in the microscopic fields. Gametocyte presence was also recorded. Genotyping samples were not collected to correct results by PCR, as a high frequency of heterologous hypnozoite activation during vivax relapse is known [26].

To access chloroquine pharmacokinetics, samples of the peripheral blood were collected either at domiciliary visits or at the day of a clinic visit and transferred to Whatman (USA) ET 31 CHR E 3MM filter papers. The whole blood concentrations of chloroquine were measured using validated high performance liquid chromatography tandem to a mass spectrometry (HPLC–MS/MS) method. This method was in accordance with Brazilian [27] and international [28] regulatory requirements for bioanalytical methods. The pharmacokinetics laboratorial analysis was conducted at the Sefar/Oswaldo Cruz Foundation, which is accredited by the Brazilian regulatory agency, ANVISA. The main pharmacokinetic parameters of C_max_, T_max_, AUC_0–t_ and AUC_0–inf_ were calculated using the software WinNonlinTM version 6.3 Pharsight and Microsoft Excel version 14.4.0. The presence of parasites with chloroquine blood levels above the 100 ng/mL threshold was considered to characterize resistance according the protocol and the WHO.

## Results

### Baseline characteristics of the study population

A total of 921 patients were screened at the Tropical Medicine Research Centre of Rondônia, Porto Velho, Brazil from April 2013 to April 2015. Eighty-eight patients who met all inclusion criteria were enrolled in the study. Patients were excluded from the study if they were unavailable to attend the study follow-up visits, resided in a remote area (41.7 %), had been administered malaria treatment within the past 63 days (21.4 %), weighted >90 kg (9.2 %), were already participating in another clinical trial (7.1 %), had a *P. falciparum* infection (3.8 %), were less than 18 years of age (2.5 %), contracted either other febrile condition or chronic disease (1 %), declined to participate (1 %), were over 70 years of age (0.4 %), or another justifiable reason (1.6 %). A flow chart is provided in Additional file 3.

Among the 88 enrolled patients, 82 (93.2 %) finished the follow-up and were included in the PP analysis. One patient withdrew the informed consent (1.1 %). Protocol deviations included one *P. falciparum* infected patient (1.1 %) and one malaria mixed infection patient (1.1 %). Three patients were lost by the follow-up visit (3.5 %). All 88 patients were included the ITT efficacy analysis and safety analysis. Qualitative and quantitative demographic population characteristics and the clinical baseline of the PP and ITT populations are presented in Tables 1 and 2.

**Table 1:** Demographic population characteristics and the clinical baseline data of the PP and ITT populations—categorical variables

Categorical variables	Absolute number and percentage (%)	
	ITT population	PP population
Total number of patients	IT = 88 (100)	PP = 82 (100)
Gender		
Male	67 (76.1)	61 (74.4)
Female	21(23.8)	21 (25.6)
Age (years)		
18–39	53 (60.2)	48 (58.50)
40–59	33 (37.5)	32 (39.0)
60–70	2 (2.3)	2 (2.5)
Weight (kg)		
>50–69	41 (46.6)	39 (47.6)
70–79	27 (30.7)	24 (29.3)
80–90	20 (22.7)	19 (23.2)
Previous malaria		
Yes	76 (86.4)	71 (86.6)
Number of previous malaria episodes		
Zero	12 (13.7)	11 (13.4)
1–5	41 (46.6)	39 (47.6)
6–10	15 (17.0)	14 (17.1)
>10	17 (19.3)	15 (18.3)
Not known	3 (3.4)	3 (3.7)
Fever during exam		
Yes	33 (37.5)	32 (39.0)

**Table 2:** Demographic population characteristics and the clinical baseline data of the PP and ITT populations—quantitative variables

	ITT		PP	
	Mean (SD)	Range	Mean (SD)	Range
Weight (kg)	70.7 (11.3)	52–90	70.7 (10.7)	51–90
Temperature (^o^C)	37.8 (1.4)	35–42	37.8 (1.4)	35–42
Parasitemia (μL)	2360	282–12,000	3353.7	282–12,000
Age (years)	38.4 (10.9)	19.3–68.6	38.2	19–68
Systolic arterial pressure (mmHg)	113 (13.2)	90–150	112.9 (13.2)	90–150
Diastolic arterial pressure (mmHg)	71.9 (9.3)	50–100	71.8 (9.6)	50–100
Number of days with symptoms prior to treatment (n = 76)	3.7 (2.2)	1–15	3.8 (2.3)	1–15

The most frequent symptoms at enrolment were fever (98.7 %), myalgia (90.9 %), chills (89.8 %) and headache (86.4 %). The concomitant treatments most reported prior to enrolment were administration of antipyretics/analgesics (46.6 %), antihypertensive agents (3.4 %) and antihistamines (2.3 %). After study initiation, 73.3 % of the population reported the administration of concomitant medications. Most of these patients took a symptomatic treatment two or three times. Non-steroidal anti-inflammatory drugs (NSAIDs) were the drugs most frequently reported concomitant medication (50.3 %). NSAIDs and antihistamines were often taken within the first 3 days after anti-malarial treatment initiation, which may be due to either to malaria symptoms or to chloroquine-related pruritus.

All treatment doses with the new chloroquine formulation and primaquine were supervised during the initial 3 days. Patients were asked about their adherence to the primaquine doses at the day 7 follow-up visit. Two patients (2.3 %) reported disruption of primaquine treatment either on day 4 or 5 but neither were classified as a treatment failure. Therefore, these patients were included in the PP analysis.

### Efficacy results

Among the 82 patients in the PP population, parasitological and clinical responses were adequate in 98.8 % (CI 95 % 93.4–100). There was one failure at day 29; however, because the protocol allows a 1-day margin for this follow-up visit, this patient was considered a late clinical and parasitological failure.

Secondary endpoints included the cure rate at day 3 (or early parasitological failure rate) and the cumulative success rate at day 28 based on the survival Kaplan–Meyer method. Early parasitological clearance 72 h after treatment initiation was evaluated in 84 patients. The success rate at day 3 was 100 %. The cumulative success rate at day 28 or the probability of remaining parasite-free at day 28 was 98.8 % (CI 95 % 91.7–99.8).

### Safety results

There were no deaths in this study, nor any other serious adverse event, as defined by GCP. No AEs led to treatment suspension. A safety analysis was conducted for the ITT population, as all patients received at least three days of chloroquine and primaquine.

A total of 380 AEs were reported during the study. The principal investigator described 304 (80 %) AEs as grade I, 74 AEs (19.7 %) as grade II, and only one (0.3 %) AE as grade III. There was no report of a grade IV AE. The number of AEs and their distribution according to the type of casualty are given in Table 3.

**Table 3:** The number of AEs and their distribution according to the type of causality

Grade	Causality Total adverse events = 380 (%)									
	Doubtful		Unlikely		Possible		Probable/likely		Total	
I	56	(14.7)	1	(0.3)	186	(48.9)	61	(16.1)	304	(80.0)
II	25	(6.6)	1	(0.3)	28	(7.4)	21	(5.5)	75	(19.7)
III	1	(0.3)	0	(0.0)	0	(0.0)	0	(0.0)	1	(0.3)
Total	82	(21.6)	2	(0.5)	214	(56.3)	82	(21.6)	380	(100.0)

Among the 214 (56.3 %) AEs classified as possibly related to the test drugs, 186 (48.9 %) were classified as grade I and 28 (7.4 %) as grade II. None of the grade III or grade IV AEs were classified as possibly related to the test drugs. There were 82 (21.6 %) events likely related to the test drugs, of which 61 (16.0 %) were classified as grade I, and 21 (5.5 %) as grade II; none were reported as grade III or grade IV. Among the 296 events possibly or likely related to the test drugs, 247 (83.4 %) were classified as grade I and, therefore, did not require any treatment.

The most frequent AEs were pruritus (56 %), headache (38.6 %), abdominal pain (35.2 %), sleep disorder (31.8 %), nauseas (28.4 %), diarrhoea (20.5 %). Vomiting, lack of appetite, cutaneous rash, muscular pain, compartmental disorder, and dizziness were reported in more than 5 % of the population. Thirty-nine patients (44.3 %) reported AEs that were classified as doubtfully related to the study drugs. AEs possibly related to the drugs were observed in 64 (72.7 %) patients, and AEs likely related to the medication were described in 49 (55.7 %) patients. A total of 74 (84 %) patients reported either an AE possibly related to or an AE likely related to the study drugs.

**Fig. 1:**
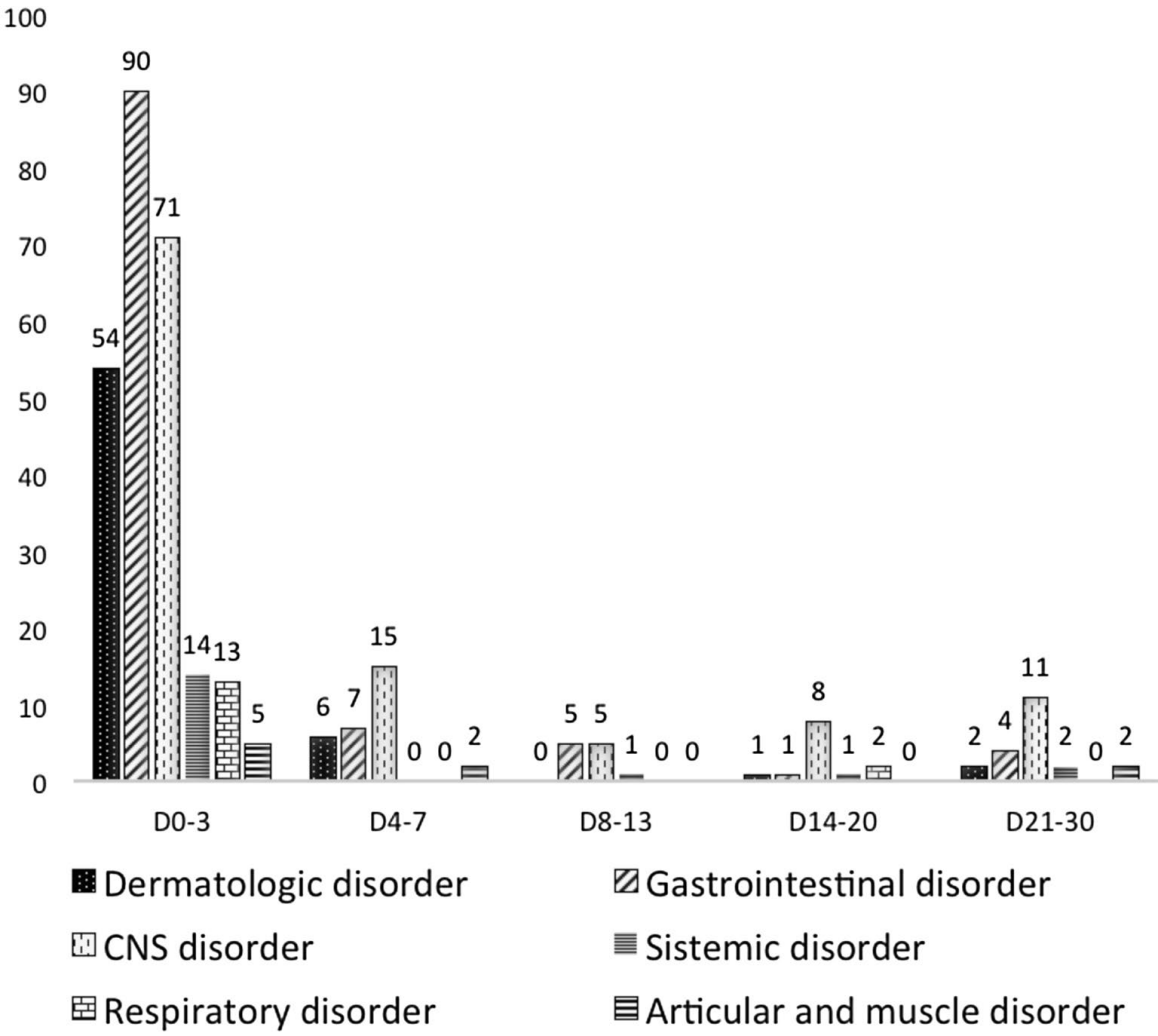
Distribution of AEs per body system disorders and follow-up visit intervals

The distribution of AEs according the main body system disorders and the follow-up visit interval is presented in Fig. 1. The frequency of all AEs sharply decreased after the third day of treatment, and further reductions were described afterwards. The only exception was the frequency of headaches, which slightly increased between the 14th and 30th days. Details of the AE counts reported per day are provided in Additional file 4.

A similar pattern was observed for the time to disappearance of symptoms. Fevers were recorded in 87 (98.9 %) patients at the time of enrolment and were present in 4 (4.5 %) patients at day 3. At the time of enrolment, 48 and 40 patients reported nausea and abdominal pain, respectively. At day 1, there was a 92.4 % decrease in reported nausea and a 68.7 % decrease in reported abdominal pain. As these symptoms are related to both the disease and treatment, they were recorded as AEs if they were not present prior to administration or they were recurrent. This overlap in symptoms may have led to an over estimation of AEs; however, it provides a conservative safety profile.

**Fig. 2:**
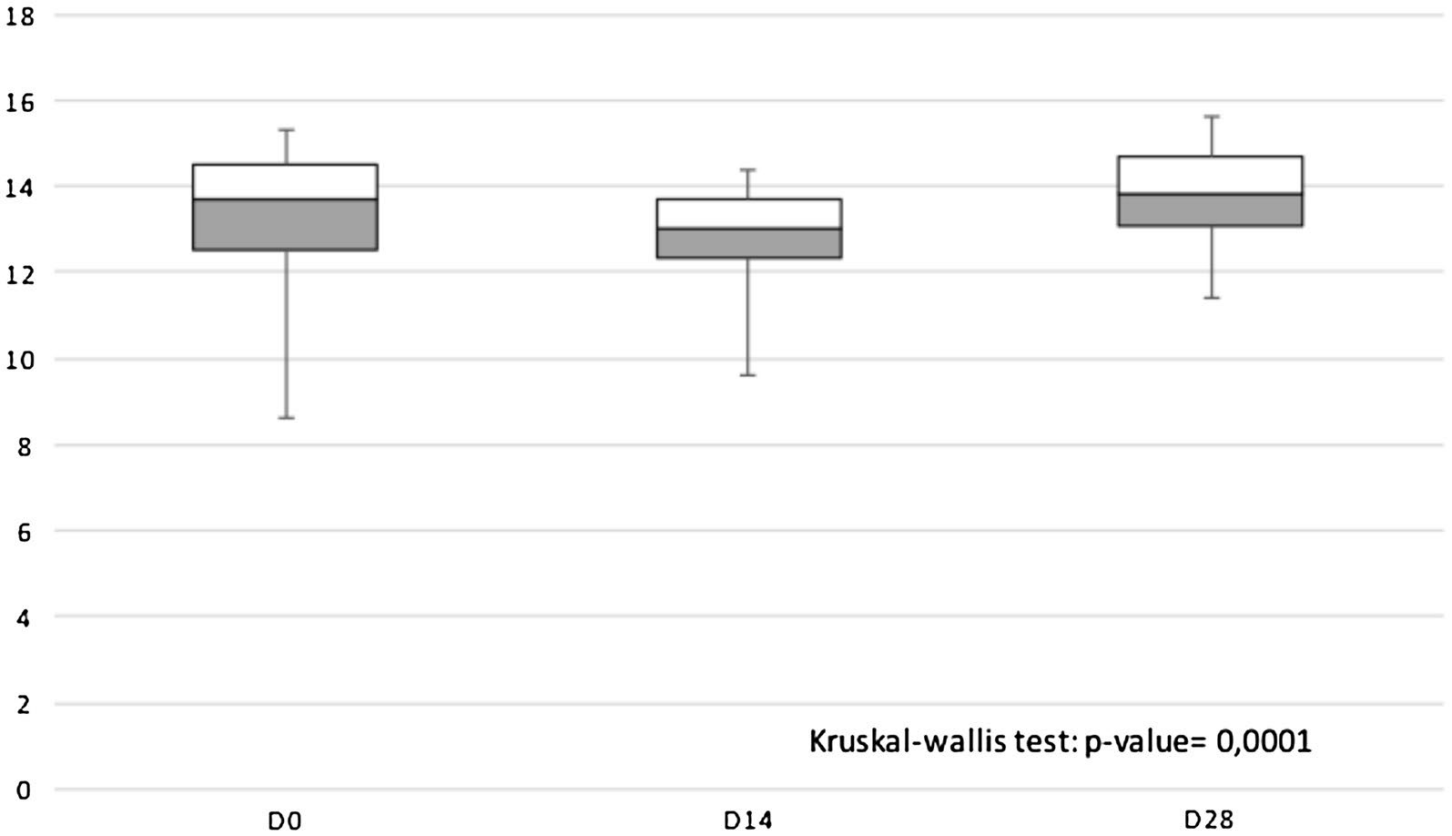
Box plots of the median haemoglobin on days 0, 14 and 28

The mean haematocrit and haemoglobin on days 0, 14 and 28 are presented in the Table 4 and Fig. 2. Although the p value was statistically significant, the variation was not clinically relevant.

**Table 4:** Median haematocrit and haemoglobin on days 0, 14, and 28

Laboratorial result	Day 0 (n = 87)		Day 14 (n = 82)		Day 28 (n = 81)	
	Median	Range (SD)	Median	Range (SD)	Median	Range (SD)
Haemoglobin	13.7	8.6–16.8 (1.5)	13.0	9.6–16.3 (1.3)	13.8	11.4–16.5 (1.1)
Haematocrit	41.1	27.8–51.5 (4.8)	39.9	32.0–48.6 (3.7)	42.2	35.8–51.5 (3.3)

### Pharmacokinetics results

**Fig. 3:**
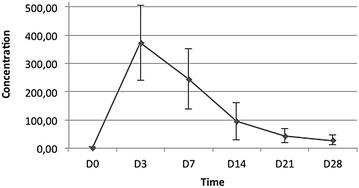
Curve of the blood concentration of chloroquine versus time

The pharmacokinetic parameters of chloroquine in whole blood dry spot samples (n = 86 patients) are presented with the mean, standard deviation, coefficient of variation, and minimum and maximum values in Table 5. Figure 3 demonstrates the mean concentrations during the study.

**Table 5:** Chloroquine pharmacokinetic parameters (n = 86)

	C_max_	T_max_	AUC_0–t_	AUC_0–inf_	Vd	Cl
	ng/mL	Days	Days (ng/mL)	Days (ng/mL)	mg/(ng/mL)	mg/days (ng/mL)
Mean	374.66	27.51	3700.43	3949.30	0.49	0.04
SD	129.57	2.09	1394.43	1431.95	0.34	0.02
CV	0.35	0.08	0.38	0.36	0.69	0.50
Minimum	122.10	14.0	690.60	826.84	0.16	0.02
Maximum	733.52	28.0	8045.24	9331.73	2.74	0.18

All patients achieved whole blood levels above 100 ng/mL. The minimum value at C_max_ was 122.20 ng/mL. The lowest value did not correspond to the patient who exhibited a clinical and parasitological failure at day 29. This patient maintained chloroquine blood levels above the minimum effective blood concentration until day 14. However, the sample collected on the failure day contained a lower concentration than 100 ng/mL and, therefore, does not characterize a resistant parasite according the protocol and the WHO. The coefficients of variation of the main pharmacokinetic parameters were high (≥0.35), which might be due to the restricted (6) sample of collection points.

## Discussion

Despite the many achievements in malaria control over the past 15 years, malaria remains a major public health problem [1]. The 18 % worldwide incidence reduction trend observed in 2014 in comparison to 2013 [1] is similar to the 19 % reduction rate in Brazil observed during the same period. In 2014, Brazil achieved the lowest incidence of malaria of the past 35 years [5]. Although progress towards falciparum elimination now represents a realizable goal that could not be foreseen 10 years ago, malaria vivax control is a more problematic issue. Vivax control also requires eliminating the appearance of early gametocytes and treatment of the extensive population of asymptomatic patients. The development of a new drug for the treatment of malaria vivax has been a known unmet need for many years, yet the primary first line of therapy has remained the same for the past 60 years in the vast majority of countries worldwide [16]. In this scenario, known interventions for improving the safe and effective use of medicine [29] may represent an additional viable strategy for controlling malaria. The proposed chloroquine–primaquine co-blister represents a subtle incremental technology that may markedly increase treatment adherence, thus eventually leading to reduced relapse rate, increased effectiveness and decreased transmission. If this intervention decreases the time to relapse, it would greatly improve the patients’ quality of life. The availability of the new packaging is pending regulatory submission and approvals.

This study presents the safety and efficacy results of a new coated chloroquine formulation; nevertheless, improving adherence, acceptability and suitability to local needs and behaviours remain clear limitations of malaria treatment, and the effectiveness for which have not yet been evaluated and remain to be proven. Further studies to are currently planned to provide this lack of data and to increase the body of evidence supporting innovative and simple approaches to improve public health. Additional pharmaceutical developments must also be pursued to address the need of children who cannot swallow tablets.

The efficacy results reported here are comparable to other recent publications in Brazil [30]. Although the efficacy was adequate at 28 days, as recommended by the WHO [23], this follow-up period may not provide the best sensitivity for evaluating vivax relapse in the clinical trial in Brazil. Further studies are ongoing and may shed light on a better time performing follow-up evaluations in malaria vivax trials. The single patient who was classified as a failure was a 29-year-old male weighting 68.4 kg with no comorbidities, low parasitaemia at day 0 (450 parasites/dL) and no parasites on day 2. Furthermore, his most recent malarial infection was due to *P. vivax* more than 1 year prior to this study, and he reported adherence to the primaquine treatment. The pharmacokinetic analysis revealed the chloroquine exposure in this patient that was similar to the other patients. The drug levels on the day of failure did not characterize the parasite as resistant. Due to the one single failure, the known high frequency of heterologous hypnozoite activation during a vivax relapse, and the lack of access to deep sequencing at the end of this study, genotyping to differentiate relapse from possible re-infection was not conducted.

In an endemic area, the probability of remaining parasite-free as long as possible is a relevant measure that contributes to patient quality of life. At day 28, the probability of the patients being parasite-free was 98.8 % in this trial. The treatments were directly observed during the first 3 days, and primaquine doses were continually adjusted per kilogram. A limitation of this study is highlighted by the fact that the initial 3 days of treatment were directly observed in this clinical trial setting, which is unlike routine health assistance in this area and may compromise the external validity of this study. On the other hand, primaquine administration was not supervised after day 3, and therefore, the actual efficacy of the results as to whether they represent the complete recommended treatment may be challenged.

Many AEs were reported in the safety results, highlighting the awareness of the study team regarding the importance of the patients’ safety profiles in a study associated with regulatory processes. Most of the AEs were mild and did not require any symptomatic treatment. The safety profile concerned treatment adherence, although it is similar to that described in literature. A review of primaquine clinical trials including safety evaluations from 11,466 patients reported a rate of serious AEs of 8.7 per 10,000 and no deaths [31]. Although chloroquine can be used for many therapeutic indications other than malaria, the AEs that occurred at a rate higher than 5 % included pruritus, headache, gastro-intestinal discomfort, and central nervous system (CNS) disorders [32]. Nevertheless, as the sample size was small and there was not a group comparison, the safety evaluation was limited. However, as the primary change in the malaria treatment was related to the formulation rather than the dosing, it can be concluded that the treatment is safe in the dose range and population studied.

## Conclusion

This study shows the safety and efficacy of a new formulation of coated chloroquine for vivax malaria treatment in Brazil. The cure rate was 98.8 % (CI 95 % 93.4–100 %) and met the WHO criteria. This study provides satisfactory evidence of the efficacy of the formulation, supporting the National Malaria Control Programme treatment guidelines. The treatment is also safe in the dose range and population studied. There is no evidence of *P. vivax* resistance to the treatment in the study population. These results support the use of a new formulation of coated chloroquine tablets in Brazil and may be the first step towards a user-friendly co-blister of chloroquine and primaquine to treat malaria vivax.

## Additional files

10.1186/s12936-016-1530-0 Co-blister preliminary design. The figure provided is an illustrative package design of the chloroquine and primaquine co-blister.
10.1186/s12936-016-1530-0 Treatment schedule. The table presents the number of tablets per drug and day according weight range.
10.1186/s12936-016-1530-0 Patient’s flow chart. The figure presents patient’s flow diagram from screening to analysis, besides the reasons for not including and study premature discontinuation.
10.1186/s12936-016-1530-0 Adverse events counts reported per day. The table provides detailed information on the absolute numbers and distribution of adverse events during the study.

## Abbreviations

ACT: artemisinin-based combination therapy
AE: adverse events
AMI/RAVREDA: Amazon Malaria Initiative/Amazon Network for the Surveillance of Anti-malarial Drug Resistance
ANVISA: National Regulatory Agency
CEPEM: Tropical Medicine Research Centre of Rondônia
CI: confidence interval
CNS: central nervous system
CONEP: Brazilian National Council on Ethics in Research
CTCAE: common terminology criteria for adverse events
FIOCRUZ: Oswaldo Cruz Foundation
G6PD: glucose-6-phosphate dehydrogenase
GCP: good clinical practice
hCG: human chorionic gonadotropin
HPLC–MS/MS: high performance liquid chromatography tandem to a mass spectrometry
ITT: intention-to-treat
NSAIDs: non-steroidal anti-inflammatory drugs
PCR: polymerase chain reaction
PP: per protocol
PVDC: polyvinylidene chloride
RDT: rapid diagnosis tests
SD: standard deviations
SEFAR: Laboratory of Pharmacokinetics
SISNEP: National System of Ethics in Research
UNIR: Federal University of Rondônia
WHO: World Health Organization
