# Supplementary material for: Safety, efficacy and pharmacokinetic evaluations of a new coated chloroquine tablet in a single-arm open-label non-comparative trial in Brazil: a step towards a user-friendly malaria vivax treatment
Source: Malar J. 2016 Sep 17;15:477. doi: 10.1186/s12936-016-1530-0 (PMC5027105; doi:10.1186/s12936-016-1530-0)
Supplement: Supplementary file 2 — 10.1186/s12936-016-1530-0 Treatment schedule. The table presents the number of tablets per drug and day according weight range. [file 12936_2016_1530_MOESM2_ESM.pdf]

Treatment schedule

**Number of tablets per drug per day**									
**Weight (kg)**	**1st Day**		**2nd Day**		**3rd Day**		**4th to 7th Days**	**8th Day**	**9th Day**
	Cq* 150 mg	Pq* 15 mg	Cq 150 mg	Pq 15 mg	Cq 150 mg	Pq 15 mg	Pq 15 mg	Pq 15 mg	Pq 15 mg
**≥ 50 - 69**	4	2	3	2	3	2	2	0	0
**70-79**	4	2	3	2	3	2	2	2	0
**80-90**	4	2	3	2	3	2	2	2	2

*Cq=Chloroquine; Pq=Primaquine
