# Supplementary figures and images for: Safety, efficacy and pharmacokinetic evaluations of a new coated chloroquine tablet in a single-arm open-label non-comparative trial in Brazil: a step towards a user-friendly malaria vivax treatment
Source: Malar J. 2016 Sep 17;15:477. doi: 10.1186/s12936-016-1530-0 (PMC5027105; doi:10.1186/s12936-016-1530-0)

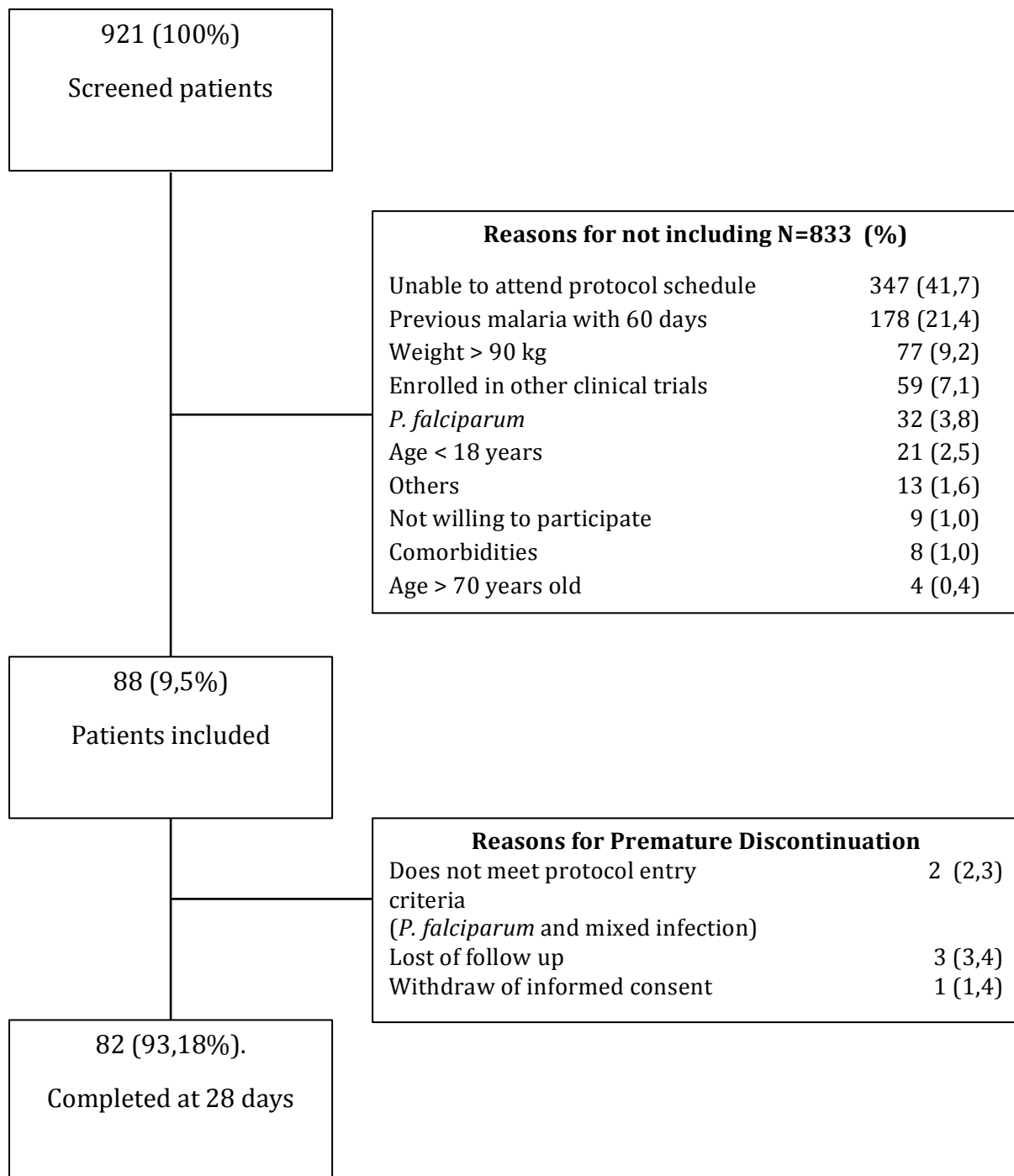

Supplement: Supplementary file 3 — 10.1186/s12936-016-1530-0 Patient’s flow chart. The figure presents patient’s flow diagram from screening to analysis, besides the reasons for not including and study premature discontinuation. [file 12936_2016_1530_MOESM3_ESM.pdf]
