# Supplementary material for: Safety, efficacy and pharmacokinetic evaluations of a new coated chloroquine tablet in a single-arm open-label non-comparative trial in Brazil: a step towards a user-friendly malaria vivax treatment
Source: Malar J. 2016 Sep 17;15:477. doi: 10.1186/s12936-016-1530-0 (PMC5027105; doi:10.1186/s12936-016-1530-0)
Supplement: Supplementary file 1 — 10.1186/s12936-016-1530-0 Co-blister preliminary design. The figure provided is an illustrative package design of the chloroquine and primaquine co-blister. [file 12936_2016_1530_MOESM1_ESM.pdf]

Frente

farmanguinhos cloroquina e primaquina

difosfato de clorquina (150 mg) e difosfato de primaquina (15 mg)

USO ORAL

EXIJA A BULA

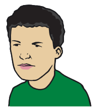

15 ANOS OU MAIS
(50 kg ou mais)

Para oito dias de tratamento

1º DIA

2º DIA

3º DIA

4º DIA

5º DIA

6º DIA

7º DIA

8º DIA

Contém 9 comprimidos de cloroquina e 16 comprimidos de primaquina

TOMAR OS COMPRIMIDOS COM LÍQUIDOS

PROIBIDA A VENDA

DISQUE SAÚDE 136

USO SOB PRESCRIÇÃO MÉDICA

SUS

Ministério da Saúde

Governo Federal

Verso

TODO MEDICAMENTO DEVE SER MANTIDO FORA DO ALCANCE DAS CRIANÇAS

Conservar em temperatura ambiente (15 a 30 ºC).
Proteger da luz e umidade.

Informações ao Paciente, Indicações, Contraindicações e Precauções: Vide Bula.

Composição: Cada comprimido revestido de Cloroquina contém 214,91 mg de difosfato de cloroquina, correspondente a 150 mg de cloroquina base.
Cada comprimido de primaquina contém 26,3 mg de difosfato de primaquina, correspondente a 15 mg de primaquina base.

Registrado por: FUNDAÇÃO OSWALDO CRUZ
CNPJ: 33.781.055/0001-35
Av. Brasil, 4365
Fabricado por: INSTITUTO DE TECNOLOGIA EM FÁRMACOS/FARMANGUINHOS
Av. Comandante Guaranys, 447 - Rio de Janeiro - RJ
Farm. Resp.: Carlos Araújo da Costa - CRF-RJ Nº 2809

M S x.xxxx.xxxx.xxx-x

Indústria Brasileira

SAC 0800 024 1692
sac@far.fiocruz.br

Fab. do Conjuto: xxxxxx
Val.: do Conjunto: xxxxx
Lote do Conjunto: xxxxx

Reserva de verniz

Frente

farmanguinhos cloroquina e primaquina
difosfato de clorquina (150 mg) e difosfato de primaquina (15 mg)
USO ORAL
EXIJA A BULA
15 ANOS OU MAIS (50 kg ou mais)
Para oito dias de tratamento
1º DIA 2º DIA 3º DIA 4º DIA 5º DIA 6º DIA 7º DIA 8º DIA
Contém 9 comprimidos de cloroquina e 16 comprimidos de primaquina
TOMAR OS COMPRIMIDOS COM LÍQUIDOS
PROIBIDA A VENDA
DISQUE SAÚDE 136
USO SOB PRESCRIÇÃO MÉDICA
SUS Ministério da Saúde Governo Federal

Verso

Nome do Paciente:______________________________

TODO MEDICAMENTO DEVE SER MANTIDO FORA DO ALCANCE DAS CRIANÇAS
Conservar em temperatura ambiente (15 a 30 ºC).
Proteger da luz e umidade.
Informações ao Paciente, Indicações, Contraindicações e Precauções: Vide Bula.
Composição: Cada comprimido revestido de Cloroquina contém 214,91 mg de difosfato de cloroquina, correspondente a 150 mg de cloroquina base.
Cada comprimido de primaquina contém 26,3 mg de difosfato de primaquina, correspondente a 15 mg de primaquina base.
Registrado por: FUNDAÇÃO OSWALDO CRUZ
CNPJ: 33.781.055/0001-35
Av. Brasil, 4365
Fabricado por: INSTITUTO DE TECNOLOGIA EM FÁRMACOS/FARMANGUINHOS
Av. Comandante Guaranys, 447 - Rio de Janeiro - RJ
Farm. Resp.: Carlos Araújo da Costa - CRF-RJ Nº 2809
M S x.xxxx.xxxx.xxx-x
Indústria Brasileira
SAC 0800 024 1692
sac@far.fiocruz.br
Fab. do Conjuto: xxxxxx
Val.: do Conjunto: xxxxx
Lote do Conjunto: xxxxx
Reserva de verniz

Frente

farmanguinhos cloroquina e primaquina

difosfato de clorquina (150 mg) e difosfato de primaquina (15 mg)

USO ORAL

EXIJA A BULA

12 A 14 ANOS
(35 a 49 kg)

Para oito dias de tratamento

1º DIA

2º DIA

3º DIA

4º DIA

5º DIA

6º DIA

7º DIA

8º DIA

Contém 7 comprimidos de cloroquina e 11 comprimidos de primaquina

TOMAR OS COMPRIMIDOS COM LÍQUIDOS

PROIBIDA A VENDA

DISQUE SAÚDE 136

USO SOB PRESCRIÇÃO MÉDICA

SUS

Ministério da Saúde

Governo Federal

Verso

TODO MEDICAMENTO DEVE SER MANTIDO FORA DO ALCANCE DAS CRIANÇAS

Conservar em temperatura ambiente (15 a 30 ºC).
Proteger da luz e umidade.

Informações ao Paciente, Indicações, Contraindicações e Precauções: Vide Bula.

Composição: Cada comprimido revestido de Cloroquina contém 214,91 mg de difosfato de cloroquina, correspondente a 150 mg de cloroquina base.
Cada comprimido de primaquina contém 26,3 mg de difosfato de primaquina, correspondente a 15 mg de primaquina base.

Registrado por: FUNDAÇÃO OSWALDO CRUZ
CNPJ: 33.781.055/0001-35
Av. Brasil, 4365

Fabricado por: INSTITUTO DE TECNOLOGIA EM FÁRMACOS/FARMANGUINHOS
Av. Comandante Guaranys, 447 - Rio de Janeiro - RJ

Farm. Resp.: Carlos Araújo da Costa - CRF-RJ Nº 2809

M S x.xxxx.xxxx.xxx-x

Indústria Brasileira

SAC 0800 024 1692
sac@far.fiocruz.br

Fab. do Conjunto: xxxxxx
Val.: do Conjunto: xxxxx
Lote do Conjunto: xxxxx

Reserva de verniz

Frente

farmanguinhos cloroquina e primaquina
difosfato de clorquina (150 mg) e difosfato de primaquina (15 mg)
USO ORAL
EXIJA A BULA
12 A 14 ANOS (35 a 49 kg)
Para oito dias de tratamento

Verso

Nome do Paciente: ______________________________

Frente

farmanguinhos cloroquina e primaquina

difosfato de clorquina (150 mg) e
difosfato de primaquina (15 mg)

USO ORAL

EXIJA A BULA

9 A 11 ANOS
(25 a 34 kg)

Para oito dias de tratamento

1º DIA

2º DIA

3º DIA

4º DIA

5º DIA

6º DIA

7º DIA

8º DIA

Contém 6 **comprimidos de cloroquina e 8 comprimidos de primaquina**

TOMAR OS COMPRIMIDOS COM LÍQUIDOS

PROIBIDA A VENDA

DISQUE SAÚDE **136**

USO SOB PRESCRIÇÃO MÉDICA

SUS

Ministério da **Saúde**

Governo Federal

Verso

Conservar em temperatura ambiente (15 a 30 ºC).
Proteger da luz e umidade.

Informações ao Paciente, Indicações, Contraindicações e Precauções: Vide Bula.

Composição: Cada comprimido revestido de Cloroquina contém 214,91 mg de difosfato de cloroquina, correspondente a 150 mg de cloroquina base.
Cada comprimido de primaquina contém 26,3 mg de difosfato de primaquina, correspondente a 15 mg de primaquina base.

Registrado por: FUNDAÇÃO OSWALDO CRUZ
CNPJ: 33.781.055/0001-35
Av. Brasil, 4365
Fabricado por: INSTITUTO DE TECNOLOGIA EM FÁRMACOS/FARMANGUINHOS
Av. Comandante Guaranys, 447 - Rio de Janeiro - RJ
Farm. Resp.: Carlos Araújo da Costa - CRF-RJ N° 2809

M S x.xxxx.xxxx.xxx-x

Indústria Brasileira

SAC 0800 024 1692
sac@far.fiocruz.br

Fab. do Conjuto: xxxxxx
Val.: do Conjunto: xxxxx
Lote do Conjunto: xxxxx
Reserva de verniz

TODO MEDICAMENTO DEVE SER MANTIDO FORA DO ALCANCE DAS CRIANÇAS

Frente

farmanguinhos cloroquina e primaquina
difosfato de clorquina (150 mg) e
difosfato de primaquina (15 mg)
USO ORAL
EXIJA A BULA
9 A 11 ANOS (25 a 34 kg)
Para oito dias de tratamento
1º DIA 2º DIA 3º DIA 4º DIA 5º DIA 6º DIA 7º DIA 8º DIA
Contém 6 comprimidos de cloroquina e 8 comprimidos de primaquina
TOMAR OS COMPRIMIDOS COM LÍQUIDOS
PROIBIDA A VENDA
DISQUE SAÚDE 136
USO SOB PRESCRIÇÃO MÉDICA
SUS
Ministério da Saúde
Governo Federal

Verso

Nome do Paciente:______________________________

TODO MEDICAMENTO DEVE SER MANTIDO FORA DO ALCANCE DAS CRIANÇAS
Conservar em temperatura ambiente (15 a 30 ºC).
Proteger da luz e umidade.
Informações ao Paciente, Indicações, Contraindicações e Precauções: Vide Bula.
Composição: Cada comprimido revestido de Cloroquina contém 214,91 mg de difosfato de cloroquina, correspondente a 150 mg de cloroquina base.
Cada comprimido de primaquina contém 26,3 mg de difosfato de primaquina, correspondente a 15 mg de primaquina base.
Registrado por: FUNDAÇÃO OSWALDO CRUZ
CNPJ: 33.781.055/0001-35
Av. Brasil, 4365
Fabricado por: INSTITUTO DE TECNOLOGIA EM FÁRMACOS/FARMANGUINHOS
Av. Comandante Guaranys, 447 - Rio de Janeiro - RJ
Farm. Resp.: Carlos Araújo da Costa - CRF-RJ N° 2809
M S x.xxxx.xxxx.xxx-x
Indústria Brasileira
SAC 0800 024 1692
sac@far.fiocruz.br
Fab. do Conjuto: xxxxxx
Val.: do Conjunto: xxxxx
Lote do Conjunto: xxxxx
Reserva de verniz

Frente

farmanguinhos cloroquina e primaquina

difosfato de clorquina (150 mg) e difosfato de primaquina (5 mg)

USO ORAL

EXIJA A BULA

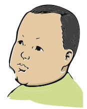

4 A 8 ANOS (15 a 24 kg)

Para oito dias de tratamento

1º DIA

2º DIA

3º DIA

4º DIA

5º DIA

6º DIA

7º DIA

8º DIA

Contém 3 comprimidos de cloroquina e 16 comprimidos de primaquina

TOMAR OS COMPRIMIDOS COM LÍQUIDOS

PROIBIDA A VENDA

DISQUE SAÚDE 136

USO SOB PRESCRIÇÃO MÉDICA

SUS

Ministério da Saúde

Governo Federal

Verso

TODO MEDICAMENTO DEVE SER MANTIDO FORA DO ALCANCE DAS CRIANÇAS

Conservar em temperatura ambiente (15 a 30 ºC).
Proteger da luz e umidade.

Informações ao Paciente, Indicações, Contraindicações e Precauções: Vide Bula.

Composição: Cada comprimido revestido de Cloroquina contém 214,91 mg de difosfato de cloroquina, correspondente a 150 mg de cloroquina base.
Cada comprimido de primaquina contém 26,3 mg de difosfato de primaquina, correspondente a 5 mg de primaquina base.

Registrado por: FUNDAÇÃO OSWALDO CRUZ
CNPJ: 33.781.055/0001-35
Av. Brasil, 4365

Fabricado por: INSTITUTO DE TECNOLOGIA EM FÁRMACOS/FARMANGUINHOS
Av. Comandante Guaranys, 447 - Rio de Janeiro - RJ

Farm. Resp.: Carlos Araújo da Costa - CRF-RJ N° 2809

M S x.xxxx.xxxx.xxx-x

Indústria Brasileira

SAC 0800 024 1692
sac@far.fiocruz.br

Fab. do Conjuto: xxxxxx
Val.: do Conjunto: xxxxx
Lote do Conjunto: xxxxx

Reserva de verniz

Frente

farmanguinhos cloroquina e primaquina

difosfato de clorquina (150 mg) e difosfato de primaquina (5 mg)

USO ORAL

EXIJA A BULA

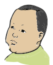

4 A 8 ANOS (15 a 24 kg)

Para oito dias de tratamento

Contém 3 comprimidos de cloroquina e 16 comprimidos de primaquina

TOMAR OS COMPRIMIDOS COM LÍQUIDOS

PROIBIDA A VENDA

DISQUE SAÚDE 136

USO SOB PRESCRIÇÃO MÉDICA

SUS Ministério da Saúde Governo Federal

Verso

Nome do Paciente:____________________
