# Supplementary material for: Safety, efficacy and pharmacokinetic evaluations of a new coated chloroquine tablet in a single-arm open-label non-comparative trial in Brazil: a step towards a user-friendly malaria vivax treatment
Source: Malar J. 2016 Sep 17;15:477. doi: 10.1186/s12936-016-1530-0 (PMC5027105; doi:10.1186/s12936-016-1530-0)
Supplement: Supplementary file 4 — 10.1186/s12936-016-1530-0 Adverse events counts reported per day. The table provides detailed information on the absolute numbers and distribution of adverse events during the study. [file 12936_2016_1530_MOESM4_ESM.pdf]

Adverse Events/ days	**0**	**1**	**2**	**3**	**4**	**5**	**6**	**7**	**9**	**10**	**11**	**13**	**14**	**16**	**19**	**20**	**21**	**22**	**23**	**25**	**27**	**28**	**29**	**30**	**Total Geral**
Itching		17	21	11				2					1					1							53
Headche	1	7	7	2	2	1	2	6	1	1	1	1	3	1		1	2	2	1		1	2			45
Abdominal pain		13	10	4			1	3	2				1				1	1							36
Disorder or difficulty sleeping	3	13	9	3				1				1	1				1								32
Nausea	2	13	9	4				1																	29
Diarrhea	3	4	5	4				1	1																18
Weakness	1	3	5	5								1	1				1				1				18
Tinnitus	1	9	4	1				1									1								17
Cough		4	6	3									2												15
Anorexia	1		5	4								1									1				12
Vomiting	1	1	5	2				1		1								1							12
Skin rashes		2	1	2				4																1	10
Muscle aches		1	2	2				2										1			1				9
Behavioral changes		4	1					1					1					1							8
Dizziness			5	1				1					1												8
Bitter mouth		1		1				1								1									4
Dyspnea								2				1											1		4
Pallor		1	1	1									1												4
Nightmare or hallucination	1	2	1																						4
Choluria			1	1				1																	3
Sore throat							1	1												1					3
Jaundice		1	1					1																	3
Somnolence		1	2																						3
Dry mouth			1	1																					2
Chill		1		1																					2

Menstrual cramps											1			1											2
Lip swelling		1						1																	2
Acrocyanosis											1														1
Afta labial				2																					1
Depression							1																		1
Toothache											1														1
Extremities swelling																		1							1
Gingival swelling						1																			1
Fever		1																							1
Tingling in hands				1																					1
Photosensitivity			1																						1
Bruise on ankle			1																						1
Hyperglycemia	1																								1
Hypertension								1																	1
Lip injury			1																						1
Nocturia				1																					1
Paresthesia of the lower lip				1																					1
Loss of balance			1																						1
1st degree burn				1																					1
Cold																					1				1
Dryness of the vaginal membrane			1																						1
Rhinitis			1																						1
Blurred vision								1																	1
Total	**15**	**100**	**108**	**59**	**2**	**2**	**5**	**33**	**4**	**2**	**1**	**8**	**12**	**1**	**1**	**2**	**6**	**8**	**1**	**1**	**5**	**2**	**1**	**1**	**380**
